# The evolution of transcriptional repressors in the Notch signaling pathway: a computational analysis

**DOI:** 10.1186/s41065-019-0081-0

**Published:** 2019-01-17

**Authors:** Dieter Maier

**Affiliations:** Institute of Genetics (240), University of Hohenheim, Garbenstr. 30, 70599 Stuttgart, Germany

**Keywords:** Notch repressors, Hairless, SHARP, KyoT2, Limpet, Prickle, Gene annotation, Evolution, Animal kingdom

## Abstract

**Background:**

The Notch signaling pathway governs the specification of different cell types in flies, nematodes and vertebrates alike. Principal components of the pathway that activate Notch target genes are highly conserved throughout the animal kingdom. Despite the impact on development and disease, repression mechanisms are less well studied. Repressors are known from arthropods and vertebrates that differ strikingly by mode of action: whereas *Drosophila* Hairless assembles repressor complexes with CSL transcription factors, competition between activator and repressors occurs in vertebrates (for example SHARP/MINT and KyoT2). This divergence raises questions on the evolution: Are there common ancestors throughout the animal kingdom?

**Results:**

Available genome databases representing all animal clades were searched for homologues of Hairless, SHARP and KyoT2. The most distant species with convincing Hairless orthologs belong to Myriapoda, indicating its emergence after the Mandibulata-Chelicarata radiation about 500 million years ago. SHARP shares motifs with SPEN and SPENITO proteins, present throughout the animal kingdom. The CSL interacting domain of SHARP, however, is specific to vertebrates separated by roughly 600 million years of evolution. KyoT2 bears a C-terminal CSL interaction domain (CID), present only in placental mammals but highly diverged already in marsupials, suggesting introduction roughly 100 million years ago. Based on the LIM-domains that characterize KyoT2, homologues can be found in *Drosophila melanogaster* (Limpet) and *Hydra vulgaris* (Prickle 3 like). These lack the CID of KyoT2, however, contain a PET and additional LIM domains. Conservation of intron/exon boundaries underscores the phylogenetic relationship between KyoT2, Limpet and Prickle. Most strikingly, Limpet and Prickle proteins carry a tetra-peptide motif resembling that of several CSL interactors. Overall, KyoT2 may have evolved from *prickle* and *Limpet* to a Notch repressor in mammals.

**Conclusions:**

Notch repressors appear to be specific to either chordates or arthropods. Orthologues of experimentally validated repressors were not found outside the phylogenetic group they have been originally identified. However, the data provide a hypothesis on the evolution of mammalian KyoT2 from Prickle like ancestors. The finding of a potential CSL interacting domain in Prickle homologues points to a novel, very ancestral CSL interactor present in the entire animal kingdom.

**Electronic supplementary material:**

The online version of this article (10.1186/s41065-019-0081-0) contains supplementary material, which is available to authorized users.

## Background

### The Notch signaling pathway

Multicellular organisms are built by a multitude of cell types that need to be determined and specified in the course of development. This often involves cell to cell communication mediated by the highly conserved Notch signaling pathway. The principal components, the receptor Notch and its ligands - for example Delta -, the signal transducing transcriptional regulator CSL (abbreviated from mammalian **C**BF1 or RBPJ, *Drosophila* **S**uppressor of Hairless and *Caenorhabditis* **L**ag1) as well as the HES (**H**airy-**E**nhancer of **s**plit) class target-genes, are found in all higher eumetazoan genomes including *Hydra vulgaris* [1–4].

Notch signaling components have been first recognized in *Drosophila melanogaster*, where mutations in the genes encoding the receptor Notch, the ligands Delta and Serrate, as well as the major antagonist Hairless were identified around 100 years ago [5–7]. This is not surprising since all these genes are haplo-insufficient, i.e. mutants develop a dominant, name giving phenotype like wing incision, thickened wing veins or loss of mechano-sensory hairs and bristles. First indications that the genes are used in a common developmental process came from the manifold genetic interactions observed in mutant combinations [8]. Later molecular genetic studies revealed the nature of the respective proteins, and allowed to propose their respective roles in Notch signal transduction in flies, and similarly in many other species as well (reviewed in: [1, 2, 9]). In fact, there is a remarkably high conservation of the core components of the Notch signaling pathway in metaozoa throughout the animal kingdom [10].

### Activation of Notch signaling

**Fig. 1:**
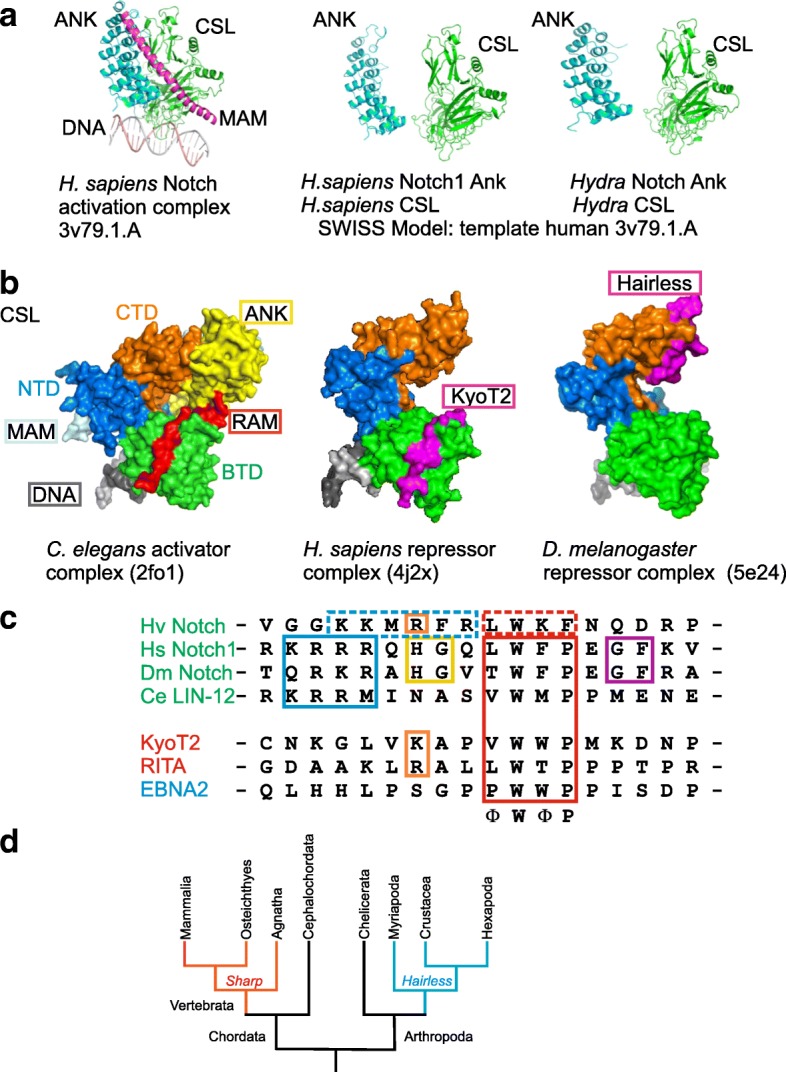
Structure of CSL transcription complexes. **a**) Structure of the human Notch activator complex on DNA (PBD ID: 3v79.1A): CSL (green), Notch 1 ANK-repeats (blue) and the kinked alpha-helical domain of MAML (magenta) (left). Structure prediction of *Hydra vulgaris* activation complex (right), depicting Notch ANK and CSL, was done by SWISS Model using 3v79.1A as template for human Notch 1 ANK and CSL (center). **b**) CSL harbors three subdomains, the N-terminal domain NTD, the beta trefoil domain (BTD) and the C-terminal domain (CTD). NTD and BTD contact the DNA (grey). Left: In the activator complex (*C. elegans* [PDB ID: 2fo1]) Notch makes contacts to the CTD with the ANK domains (yellow), and to the BTD with its RAM domain (red). Mam (light blue) contacts BTD and ANK. Middle: The CSL-ID of KyoT2 (pink) (PDB-ID: 4J2X) makes very similar contacts as RAM with the BTD. Right: In contrast, contacts between fly Hairless (pink) and Su(H) are restricted to the CTD only (PDB ID: 5E24). **c**) Sequence comparison of the CSL-interacting domain from KyoT2, RITA and EBNA2 with RAM domains of Notch [*Hydra vulgaris* (Hv Notch), *Homo sapiens* (Hs Notch1), *Drosophila melanogaster* (Dm Notch), *Caenorhabditis elegans* (Ce LIN-12)]. Note lack of the typical ΦWΦP motif in *Hydra* Notch (Φ, any hydrophobic residue). **d**) Simplified phylogenetic tree of chordates and arthropods. Red: branches with SHARP coding genes, blue: with *Hairless* coding genes

Simplified, Notch signaling activity is triggered by the binding of the membrane tethered ligand on one cell to the receptor Notch of the adjacent cell, resulting in the release of the Notch intra-cellular domain (NICD). NICD itself allows assembly of a transcription activator complex on Notch target gene promoters, together with CSL and the co-activator Mastermind (Mam) (Fig. 1a), as well as additional chromatin activators like histone acetyltransferase (p300, PCAF), histone demethylase (LSD1/CoREST) and histone ubiquitin-ligase (Bre1) (reviewed in: [11–13]). CSL contains three conspicuous domains, a N-terminal domain NTD, a beta-trefoil domain BTD, and a C-terminal domain CTD (Fig. 1b). Whereas NTD and BTD make sequence-specific DNA-contacts, NICD contacts both BTD and CTD, the former with the so-called RAM (**R**BP-Jκ-**a**ssociated **m**olecule) domain, and the latter with the Ankyrin (ANK) repeats (Fig. 1b) (reviewed in: [11–15]).

The activation process has been studied experimentally in several organisms, concentrating mostly on the nematode *Caenorhabditis elegans*, the insect *Drosophila melanogaster* and several vertebrate species including *Danio rerio* (zebra fish), clawed frog *Xenopus laevis*, *Mus musculus* and *Homo sapiens* (reviewed in: [16]). Interestingly, the co-activator Mam shows little conservation [17]. Mam is part of the activator complex, contacting CSL and NICD with a bent alpha helix consisting of about 60 residues (Fig. 1a,b). Although Mam proteins are rather large, this alpha helix is the only common character between proteins from fly, worm and mammals (reviewed in: [11, 14, 17]). No sequences encoding a Mam-like protein were identified in the genome of the cnidarian *Hydra vulgaris* to date, including my own attempts. The few alignments not only lack an alpha helix, but also the conserved residues required for trimeric complex formation [18]. However, a Mam like gene it is present in the sea anemone *Nematostella vectensis* (Anthozoa) [10].

### Competition for the binding of CSL’s beta trefoil domain

Whereas the principles of Notch target gene activation are well conserved, repression mechanisms are of bewildering variance, and have been primarily studied in mammals and *Drosophila*. CSL is central to Notch target gene regulation, be it activation or repression. Two principle mechanisms of repression apply (Fig. 1b) (reviewed in: [9, 13, 19]): Firstly, a competition of proteins with NICD for the binding of CSL, thereby decreasing availability of CSL for Notch and hence, activation levels of the target genes. Competition may be direct or indirect as outlined below. Secondly, assembly of a repressor complex on Notch target gene promoters, resulting in silencing of the target genes. Repressor complex formation involves CSL that hence can act as a molecular switch depending on the recruitment of co-activators versus co-repressors [19, 20]. In addition, recruitment of chromatin modifiers, notably histone deacetylases (HDACs, SMRT/Sin3A) and histone chaperones (e.g. Asf1), results in chromatin inactivation (reviewed in: [13]).

**Fig. 2:**
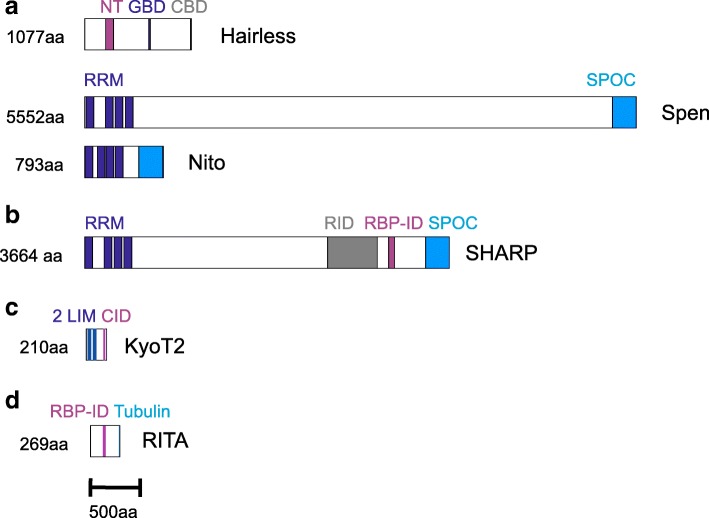
Cartoons of validated Notch repressors. Notch repressor proteins characterized in *Drosophila* and mammals are shown to scale. Protein size is given in amino acids (aa). **a**) *Drosophila melanogaster*. Upper panel displays H protein structure with the Su(H) interacting NT box (NT), the Groucho binding domain (GBD) and the C-terminal binding protein binding domain (CBD). Split ends protein (Spen, center panel) and Spenito (Nito, lower panel) contain four RNA recognition motifs (RRM) at the N-terminus, and a Spen paralog and ortholog C-terminal (SPOC) domain. **b**) *Homo sapiens*. SHARP protein belongs to the Spen protein family; it contains in addition to the RRM and the SPOC domain also a receptor interacting domain (RID), and the RBPJ interacting domain (RBP-ID). **c**) *Mus musculus*. KyoT2 is characterized by two complete LIM domains and the CSL interacting domain (CID) **d**) *Homo sapiens*. RITA protein contains a central RBP interacting domain (RPB-ID) and a Tubulin binding motif at the C-terminus

The direct competition mechanism appears to be restricted to mammals. It specifically involves the RAM-BTD interaction (Fig. 1b): the RAM domain of NICD, including a tetra-peptide motif ΦWΦP (Φ, any hydrophobic residue), makes hydrophobic contacts with a nonpolar pocket of the BTD (reviewed in: [12, 15]) (Fig. 1b, c). Several proteins have been identified to exploit this interaction site, including repressors of Notch activation and the EBNA2 (**E**pstein-**B**arr **n**uclear **a**ntigen **2**) viral activator hijacking the process [21–25]. Structural analyses revealed stunning similarities between RAM and repressor binding; notably the tetra-peptide ΦWΦP specific contacts are highly conserved (Fig. 1c). Primary examples of CSL-repressor complexes include *Mus musculus* KyoT2 or *Homo sapiens* RITA (RBPJ interacting and tubulin associated) (Figs. 1b, c, 2) [19, 24, 25]. RITA is a small, tubulin-associated protein that not only competes with NICD for CSL binding, but results in CSL depletion by its nuclear export [26]. RITA is highly conserved in deuterostomes including Placozoa but, interestingly, not found in insects [26]. Because its evolution has been examined already, it was not included in this study. Despite the in vitro binding of human RITA to *Drosophila melanogaster* Su(H) and tubulin, no repression of Notch signaling activity was observed in vivo, indicating that this mechanism of Notch regulation does not apply to flies [27]. In contrast to RITA, little is known about the evolution of mouse KyoT2 (Fhl1C in human), one of three splice variants of KyoT (named also Fhl1, four and a half LIM domains). Each LIM domain comprises two tandemly repeated zinc finger domains, separated by a two-amino acid hydrophobic linker, thought to serve protein-protein interactions [28]. The KyoT2 splice variant is smaller, encoding just two and a half LIM domains followed by the CSL interacting domain (CID) encoded by the specific exon [21] (Fig. 2). Apart from the common tetra-peptide ΦWΦP motif, KyoT2 shares not structural similarity with RITA.

### Repressor complex formation on Notch target genes

Repressor complex formation has been investigated both in vertebrates and *Drosophila* (Fig. 1d). It seems to be the primary mechanism of Notch target gene repression in flies, where it has been studied experimentally in detail. A major antagonist of Notch signaling in *Drosophila melanogaster* is named Hairless (H), which binds to the fly CSL ortholog Suppressor of Hairless [Su(H)] (Figs. 1a, 2a). By recruitment of the two general co-repressors Groucho (Gro) and C-terminal binding protein (CtBP), Hairless assembles a repressor complex on Notch-target gene promoters, thereby eventually silencing gene activity [29–32] (reviewed in: [9]). The structure of the Su(H)-H repressor complex has been solved recently at the molecular level and confirmed experimentally in vivo [33]. Hairless binds to the CTD of Su(H) in a unconventional way: The Su(H) binding domain of Hairless forms beta-strands that slide deeply into the hydrophobic core of the CTD, distorting the structure in a way that excludes binding of Notch ANK domains [33] (Fig. 1b).

Based on manifold genetic data revealing a quantitative antagonism between Notch and Hairless, a threshold model was postulated with Hairless competing with NICD for Su(H) binding in the cytoplasm [8, 34, 35]. As we know now, both Hairless and NICD bind Su(H) with similar affinities to form transcriptional repressor and activator complexes, respectively [33, 36, 37]. Moreover both, Hairless and NICD, are involved in nuclear shuttling of Su(H) [29, 38–41]. Conservation of Hairless has been extensively studied (Fig. 1d): Orthologs are present in all insects studied to date as well as in some members of the subphylum Crustacea [9, 42–44]. The *D. melanogaster* Hairless protein consists of 1077/1059 residues, however, is significantly smaller in more distant species like the honeybee *Apis mellifera* (392 aa) or the water flea *Daphnia pulex* (448 aa). Common to all Hairless orthologs is the Su(H) binding domain (SBD), that can be subdivided into a N-terminal NT and a C-terminal CT box, with the former being sufficient for Su(H) contacts (~ 37 residues). In addition, they all share the Gro binding domain GBD (~ 9 aa) and the CtBP binding domain CBD (~ 10 aa), as well as three potential nuclear localization signals (NLS) and one predicted nuclear export signal (NES) [37, 43–45]. The three binding domains are well conserved, notably the NT box, which specifies Hairless orthologs (Fig. 2).

Repressor complex formation in *Homo sapiens* involves SHARP (**S**MRT/**H**DAC1 **a**ssociated **r**epressor **p**rotein), also called MINT in *Mus musculus* (**M**SX-2 **i**nteracting **n**uclear **t**arget), suggested to be the functional homolog of Hairless [46–48]. In contrast to the above repressors, SHARP not only binds to the BTD of CSL but also to the CTD, and recruits CtBP and CtIP as additional co-repressors to silence Notch target gene expression [48, 49]. The SHARP protein is very large (3664 amino acids in human) and contains four structural motifs, the RRM (RNA recognition motif) at the N-terminus and the SPOC (Spen paralog and ortholog C-terminal) domain at the C-terminus, the RID (**r**eceptor **i**nteracting **d**omain) and the RBPJ interacting domain (RBP-ID) in the center (Fig. 2b). SHARP is also named SPEN after the *D. melanogaster* protein Split ends (Spen), which shares the RRM and SPOC domains, however, lacks the RID and RBP-ID of SHARP [50–52]. Accordingly, both activating and repressive genetic interactions have been described between *spen* and members of the Notch pathway in the fly [53], but no direct physical protein interactions.

Regarding KyoT2, experimental data on Notch repression have been gathered in addition to structural analyses of the repressor complex [21, 24]. Evolutionary data, however, are lacking so far.

Taken all together, the knowledge on the negative regulation of Notch target genes is rather limited. It remains largely restricted to model systems, where Notch signaling has been studied experimentally, as well as to humans, where Notch regulation is of particular medical interest. And as outlined above, various mechanisms of regulation appear to exist in the animal kingdom used by some but not all clades. In the past years, sequence information for many more species has become available, allowing searches for possible repressor proteins outside of insects and vertebrates. Therefore, a search of the genome databases was performed, concentrating on the following questions: a) is there an evolutionary bond between the various repression mechanisms and b) are there homologues of the known repressors outside of arthropods or vertebrates?

## Results

### Repression complex formation in arthropods

In contrast to the extremely high conservation of CSL proteins, the *Hairless* gene turned out to be evolving fast [9, 54]. Accordingly, evolutionary comparisons restricted Hairless orthologs to the class of Insecta for quite some time [42, 43]. Recently we identified *Hairless* orthologs outside of insects in two classes of crustacean, in *Daphnia pulex* (water flea, Branchiopoda) as well as in *Litopenaeus vannamei* (whiteleg shrimp, Malacostraca). Moreover, functional conservation of *Daphnia pulex* Hairless was confirmed experimentally in transgenic *D. melanogaster* [44]. Further searches identified *Hairless* orthologs in the crustacean *Triops cancriformis* (Tadpole shrimp) and potentially also in the centipede *Strigamia maritima* (European centipede). *Triops* is described as the oldest still living animal fossil (with about 300 million years of evolution), and *T. cancriformis* may be morphologically unchanged for the past 180 million years. *Strigamia* belongs to subphylum Myriapoda that separated around 500 million years ago from dipteran flies (Fig. 1d). However, *Hairless* orthologs are not present in all Arthropoda. For example, no significant sequence conservation to Hairless could be detected in Chelicerata (550 mio years) (Fig. 1d) [55] or more distant clades.

### Hairless in *Triops cancriformis* and Strigamia maritima

**Fig. 3:**
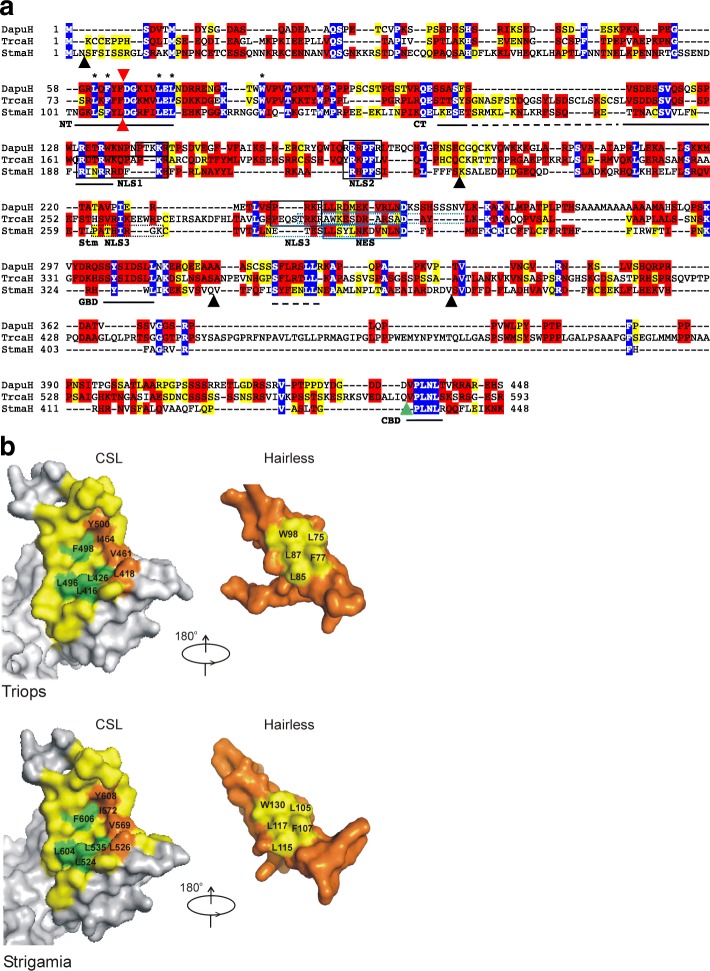
Hairless orthologs from Triops and Strigamia. **a** Alignment of predicted Hairless orthologs from *Triops cancriformis* (TrcaH) and *Strigamia maritima* (StmaH) with the *Daphnia pulex* (DapuH) ortholog. *Daphnia* and *Triops* Hairless share the characterized functional domains, the Su(H) binding domain SBD which is subdivided into NT and CT box, the Groucho binding domain GBD, and the CtBP binding domain CBD. Only NT box and CBD are clearly discernable in *Strigamia* Hairless. The CT box and the GBD may be present with weak conservation only (dashed lines). Notably, residues known to contact the Su(H) CTD in *D. melanogaster* are identical in all the species (*). High confidence NLS and NES sequences are boxed, those of low confidence are dotted. Red arrowheads indicates an intron shared by all three genes. Black and blue arrowheads depict intron positions specific to *Strigamia* and *Triops Hairless* genes, respectively. Identical residues are marked in blue, highly conserved in red, conserved in yellow. **b** Structure predictions of the Notch repressor complexes from *Triops* and *Strigamia*, respectively, using SWISS Model and template PDB ID: 5E24 of the *Drosophila* complex. Left CSL CTD and right Hairless interacting domain. Potentially interacting residues are highlighted in yellow in Hairless, and green and orange in CSL

The predicted *Hairless* ortholog of *Triops cancriformis* (*TrcaH*) (Fig. 3a) has two introns at positions also found in *D. melanogaster Hairless* (*DmH*). *TrcaH* encodes a highly basic protein (pI 10.3) of 593 amino acids (Additional file 1: Dataset 1) that shares the well defined functional domains, the NT and CT boxes of the SBD (Suppressor of Hairless binding domain), the GBD (Groucho binding domain) and CBD (CtBP binding domain). All the amino acids in the NT box demonstrated to be necessary for Su(H) binding in *D. melanogaster*, are 100% conserved [33] (Fig. 3a). Accordingly, they are at identical positions as the ones in *D. melanogaster* in the modeled structure (Swiss Model) (Fig. 3b). Moreover, three basic stretches can be identified, which may function as nuclear localization signals (Fig. 3a). A nuclear export signal predicted in *Drosophila* Hairless by NetNES 1.1 Server [45], however, has low scores, and an alignment to other NES sequences of *D. melanogaster* or *D. pulex* reflect poor conservation. The flanking series of basic amino acids may serve as NLS, thereby resembling the NLS/NES module of Hairless orthologs in other species (Fig. 3a).

With *Strigamia maritima*, a first Myriapod genome sequence has become available. Deposited in flybase (www.flybase.org) five potential orthologous *Hairless* genes are predicted by OrthoDB for *S. maritima*. However, examination of the protein sequences reveals only for one of them (SMARO13018 with 478aa) similarities to the NT box of the *D. melanogaster* Hairless protein (Additional file 1: Dataset 1). Since neither GBD nor CBD are contained within the predicted SMARO13018 protein, the complete contig of the whole genome shotgun sequence was analyzed for further interacting motifs. Indeed, a well conserved CBD motif was found about 22 kb downstream of the NT box (Additional file 2: Figure S1). If this sequence is in fact part of the *Strigamia Hairless* ortholog (*StmaH)*, the *StmaH* gene must contain more than 20 kb intron sequences (Additional file 2: Figure S1). Moreover, a stop codon shortly after the CBD coding sequence is extremely likely, albeit not present in the current database sequence, based on the high AT content of the flanking sequences typical for untranslated trailer sequences (Additional file 2: Figure S1).

In this case, the predicted StmaH protein has about 448 residues (Fig. 3a) (Additional file 1: Dataset 1). Since there are no other landmarks in the peptide sequence, this annotation is highly speculative. However, the StmaH protein has a well conserved NT box with 100% identical Su(H) interacting residues, it has a CBD and several basic regions that could serve as nuclear localization signals, and it is overall very basic typical of Hairless proteins with a pI of 10.25 (DmH, pI 10.4). A GBD might be present, however, shares little similarity to the GBD of other Hairless orthologs. A NES might exist based on the alignment with *Daphnia* Hairless, however, without a flanking NLS (Fig. 3a). The modeled structure predicts a repressor complex in *Strigamia* that convincingly matches that of the other arthropod species (Fig. 3b).

It was not possible to identify *Hairless* orthologs in genome sequences from spiders or lower arthropods (Fig. 1d). The rather weak conservation between *Strigamia* Hairless and Hairless proteins from Crustacea and Insecta may be taken as an indication that *Hairless* was firstly introduced about 550 million years ago after the Chelicerata-Myriapoda radiation (Fig. 1d).

### Repression of Notch target genes in vertebrates

#### The evolution of SHARP/SPEN

SHARP (or MINT) has been proposed to serve as functional homolog of Hairless in vertebrates for repressor complex assembly on Notch target genes [13]. SHARP belongs to the group of SPEN proteins characterized by a RRM and a SPOC domain (Fig. 2b). SPEN proteins come in two flavors, a very large form containing non-conserved internal sequences characterized by stretches of poly-amino acids (mainly Gln, His, Ser, Glu, Lys, Arg), and a tiny form named Spenito (Nito) in *D. melanogaster* (Fig. 2b) [52, 56]. DNA sequences potentially encoding SPEN protein homologues could be identified in all metazoan animals including sponges and Placozoa based on the good conservation of these two motifs, independently of whether using *D. melanogaster* Spen or Spenito or human SHARP as query. The sequences are conserved well enough that a tblastn search under almost standard conditions in NCBI database detects these domains even using whole genome shotgun sequences as template. If available, the mRNA sequences have been analyzed. However, in many cases the coding regions have been only predicted. For most species it was possible to define both, SPEN and NITO like homologues, suggesting that there is normally at least one of each present in the genome of higher animals.

Vertebrate SPEN proteins differ from all the others, however, in that they contain two additional motifs not found elsewhere (Figs. 1d, 2b) [47, 57]. One, abbreviated RID, represents the nuclear hormone receptor interacting domain, the other is named RBPJ or CSL interacting domain (RBP-ID) and mediates SHARP/MINT binding to RBPJ to allow its function as direct repressor of Notch signaling activity [13]. *D. melanogaster* Spen, however, lacks a respective Su(H) binding domain and hence repression capacity.

**Fig. 4:**
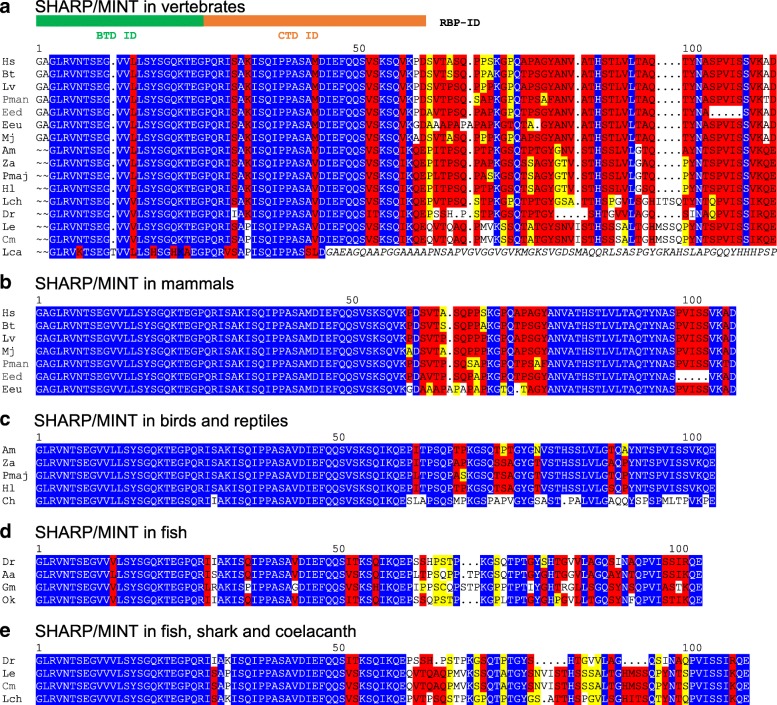
Comparison of SHARP/MINT proteins in vertebrates. Comparison of SHARP/MINT orthologues in vertebrates; shown is the alignment of the RBP-ID and C-terminally adjacent amino acids. Identical residues are marked in blue identical, highly conserved in red, conserved in yellow. **a** Alignment over the whole vertebrate tree, **b**) alignment of mammals, **c**) alignment of birds and reptiles, **d**) alignment of fish, **e**) alignment of zebra fish with chondrichthyes (cartilaginous fish) and coelacanth. Human RBP-ID, shown to interact with CSL BTD and CSL CTD, is highly conserved in all vertebrates except the arctic lamprey, where it ends a bit short. Also C-terminally adjacent sequences except of lamprey (italic) are conserved, which becomes apparent in the comparison of the group specific alignments. The rattlesnake, however, is distinctly different from alligator and birds in this region. Mammals: Hs: *Homo sapiens* (man), Bt: *Bos taurus* (cattle), Lv: *Lipotes vexillifer* (chinese river dolphin), Mj: *Manis javanica* (malayan pangolin), Pman: *Peromyscus maniculatus* (great tit), Eed: *Elephantulus edwardii* (cape elephant shrew), Eeu: *Erinaceus europaeus* (european hedgehog), Reptile and birds: Am: *Alligator mississippiensis* (alligator), Za: *Zonotrichia albicollis* (sparrow), Pmaj: *Parus major* (great tit)*,* Hl: *Haliaeetus leucocephalus* (bald eagle), Ch: *Crotalus horridus* (timber rattlesnake), Fish: Dr.: *Danio rerio* (zebrafish), Aa: *Anguilla Anguilla* (eel), Gm: *Gadus morhua* (ghostshark), Ok: *Oncorhynchus kisutch* (coho salmon)*,* Le: *Leucoraja erinacea* (little skate), Cm: *Callorhinchus milii* (ghostshark), Lca: *Lethenteron camtschaticum* (lamprey), Lch: *Latimeria chalumnae* (coelacanth) See also Additional file 3 Dataset 2 for common names

A screen for SHARP proteins with RBP-ID revealed that all vertebrates including the distant arctic lamprey (*Lethenteron camtschaticum*) possess this domain (Fig. 4a-e). However, the RBP-ID is not found in the available sequences of the amphioxus (lancelet) genome, or in any other of the searched sequences outside of vertebrates (Fig. 1d). The RBP-ID shows remarkable conservation: approximately 50 residues are very well conserved up to the distantly related arctic lamprey, whereas the C-terminal sequences are more variable, and that of lamprey could be not aligned to the other orthologs (Fig. 4a). In *C. elegans*, two SPEN like proteins, named DIN-1S and DIN-1 L, are formed by alternative splicing. The former contains an experimentally defined nuclear hormone receptor interacting domain, which however shares no sequence homology to the receptor interaction domain RID of vertebrates [58]. Both DIN-1 splice variants, however, lack homology to RBP-ID.

**Fig. 5:**
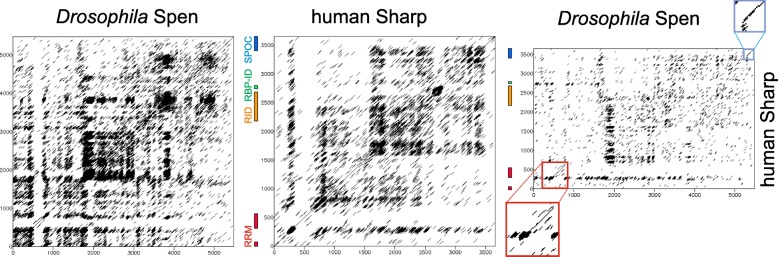
Dotplot analysis of Spen and SHARP. The dotplot analysis of *Drosophila* Spen and human SHARP shows typical repetition patterns that reflect the accumulation of single amino acids. A dotplot comparison *Drosophila* Spen with human SHARP reveals clear similarities only in the RRM and SPOC domains shown enlarged (red box, RRM and blue box, SPOC domain). The RID (orange) and RBP-ID (green) domains in SHARP are indicated as well

The sheer size of SPEN proteins complicates direct comparisons. A dotplot analysis of *Drosophila* Spen and human SHARP revealed many repetitive spots, reflecting the poly-amino acids stretches but not repeated sequence stretches (Fig. 5). Moreover, just two convincing alignments, the N-terminal RRM and the C-terminal SPOC domain, were uncovered. Neither the receptor interacting domain nor the RBPJ interacting domain of SHARP aligned convincingly to any sequence within Spen (Fig. 5). This leads to the conclusion that these motifs are specific to vertebrates, introduced into their genomes about 600 million years ago (Fig. 1d).

#### The evolution of KyoT2

KyoT2 is a well characterized repressor of Notch signaling in mammals [21, 59, 60]. KyoT2 derives from a specific splice form of KyoT, a member of the Fhl1-family of proteins that contain **f**our and a **h**alf **L**IM domains [21]. KyoT2, however, is characterized by just two and a half LIM domains followed by a short tetra-peptide motif (VWWP) that strongly binds to the BTD of RBPJ, called CID (C- terminal interaction domain) (Figs. 1b, c, 2c) [21, 24]. Based on structural analysis, the tetra-peptide motif contacts CSL similarly to the one of Notch RAM or of RITA (Fig. 1c) [21, 24, 25].

**Fig. 6:**
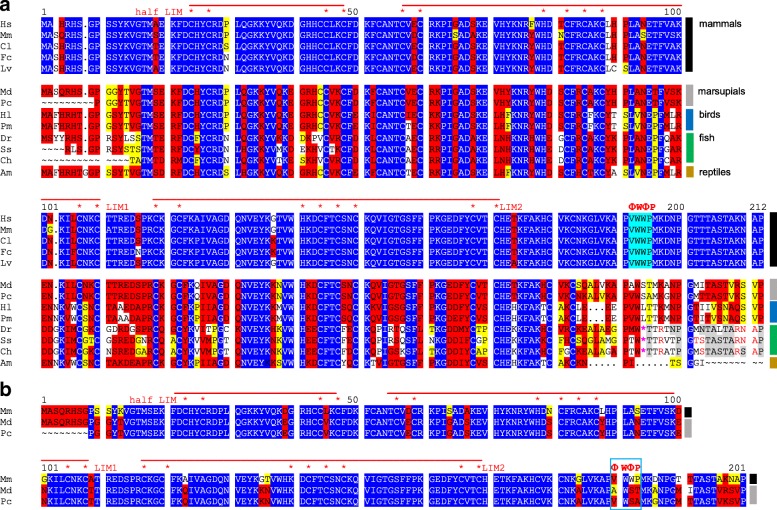
Alignment of KyoT2 homologues from vertebrates. **a** Only placental mammals contain a KyoT2 protein with conserved CSL binding motif (ΦWΦP, cyan). Already in marsupial (koala *P. cinereus* and opossum *M. domestica*) or birds (eagle *H. leudocephalus* and tit *P. major*) the motif is changed, but an open reading frame still remains. In fish, a stop codon (*) occurs exactly within the motif, however, sequences C-terminally translate into many conserved residues (grey) compared to mammals. All proteins consist of two and a half LIM domains (red lines), each containing two tandemly repeated zinc fingers. Asterisks indicate zinc binding residues. Blue are identical, red are highly conserved and yellow are related residues. Note changes in marsupials. **b** Alignment of mouse KyoT2 with marsupial koala and opossum. Note high overall conservation. The ΦWΦP tetra-peptide motif is highlighted Placental mammals (black box): Hs: *Homo sapiens* (man), Mm: *Mus musculus* (mouse), Cl: *Canis lupus* (wolf), Fc: *Felis catus* (cat), Lv: *Lipotes vexillifer* (chinese river dolphin); Marsupials (grey box): Pc: *Phascolarctos cinereus* (koala), Md: *Monodelphis domestica* (opossum). Birds (blue box): Hl: *Haliaeetus leucocephalus* (bald eagle), Pm: *Parus major* (great tit). Fish (green box): Dr.: *Danio rerio* (zebrafish), Ss: *Salmon solar* (atlantic salmon), Ch: *Clupea harengus* (herring). Reptiles (brown box): Am: *Alligator mississippiensis*.

LIM-domain containing proteins are present throughout the animal kingdom. KyoT2-like proteins that contain the CID, however, are restricted to placental mammals: here the tetra-peptide VWWP motif is 100% conserved (Fig. 6a). Already in marsupials this motif is changed [AWST in Opossum (*Monodelphis domestica*), VWSA in Koala (*Phascolarctos cinereus*)] although located at an identical position according to sequence alignments (Fig. 6a, b). Whether marsupial KyoT2 proteins can bind their cognate CSL homologues remains to be determined, however it seems unlikely, as both miss the conserved proline residue at position four important for RAM-CSL contacts [61]. Outside of mammals, no KyoT2 homolog with a typical CID was identified so far (Fig. 6 a). The analyzed fish sequences (for example the zebrafish *Danio rerio*) contain a stop codon in its place. Computational translation of 3′ adjacent codons, however, reveals considerable conservation beyond the stop (Fig. 6 a). This may be taken as hint for an introduction of either the CID coding sequences or the stop codon in the recent evolution of KyoT genes.

**Fig. 7:**
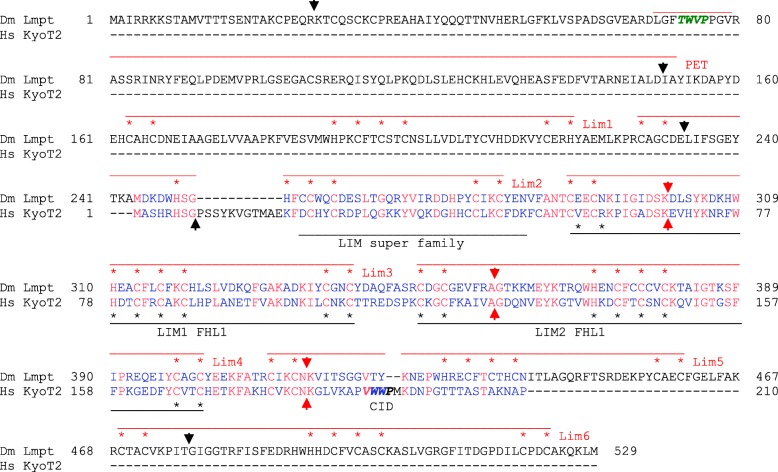
*Drosophila* Limpet is a homolog of human KyoT2. *D. melanogaster* Limpet protein harbors a PET domain, followed by six LIM domains. The alignment with human KyoT2 (also named Fhl1C) reveals surprising similarity between the LIM domains of KyoT2 (black labelling) and LIM domains two to four of *Drosophila* Limpet (isoform PN, red labelling). Intriguingly, three identical intron positions are present within this region (red arrows). Black arrows mark intron positions specific to *Limpet*. The CSL binding domain of KyoT2 (CID, italics) is not present in Limpet at a corresponding position. A similar tetra-peptide motif TWVP (italic green), however, is located at the N-terminus of the PET domain. Red residues are identical between the two proteins, blue are not conserved, black absent in one of the two species

In search for a KyoT2 homolog in *D. melanogaster,* cDNA sequences were screened using tblastn. This returned Limpet (Lmpt), a protein containing six LIM domains as well as a PET domain (named for **P**rickle, **E**spinas and **T**estin, where this domain was originally defined) at the N-terminus (Fig. 7) [62, 63]. Apart from these extra domains, Lmpt and KyoT2 are remarkably similar in their LIM domains. What is even more compelling is the fact that both genes share introns at identical positions in the LIM domain coding sequences, a clear evidence of their phylogenetic relationship (Fig. 7, Fig. 10b). Similar to KyoT, *Lmpt* has several splice variants, but none contains a CID at a position corresponding to that in KyoT2. PET domain containing splice variants, however, feature a ΦWΦP tetra-peptide motif TWVP similar to the CID of KyoT2 (Fig. 7). Whether this Lmpt-motif can provide respective CSL interactivity, remains to be shown.

**Fig. 8:**
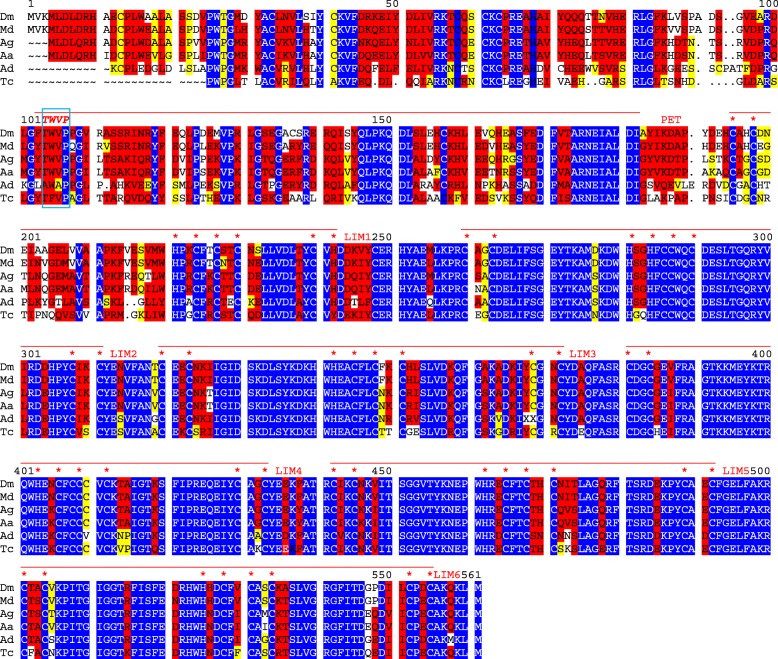
Limpet orthologs in insects. Limpet orthologs are only found in higher insects. Highest conservation is seen in the LIM domains with many identical residues (blue), whereas the PET domain and the N-terminus are less well conserved. The tetra-peptide Lmpt-motif TWVP (italic, boxed) is present in all aligned sequences at identical position, with conservative variations seen in *Tribolium castaneum* (Tc) and *Apis dorsata* (Ad). Flies, Dm: *Drosophila melanogaster*, Md: *Musca domestica*, Ag: *Anopheles gambiae*, Aa: *Aedes aegypti*, Bee, Ad: *Apis dorsata*. Beetle, Tc: *Tribolium castaneum* (see also Additional dataset 2 for common names)

**Fig. 9:**
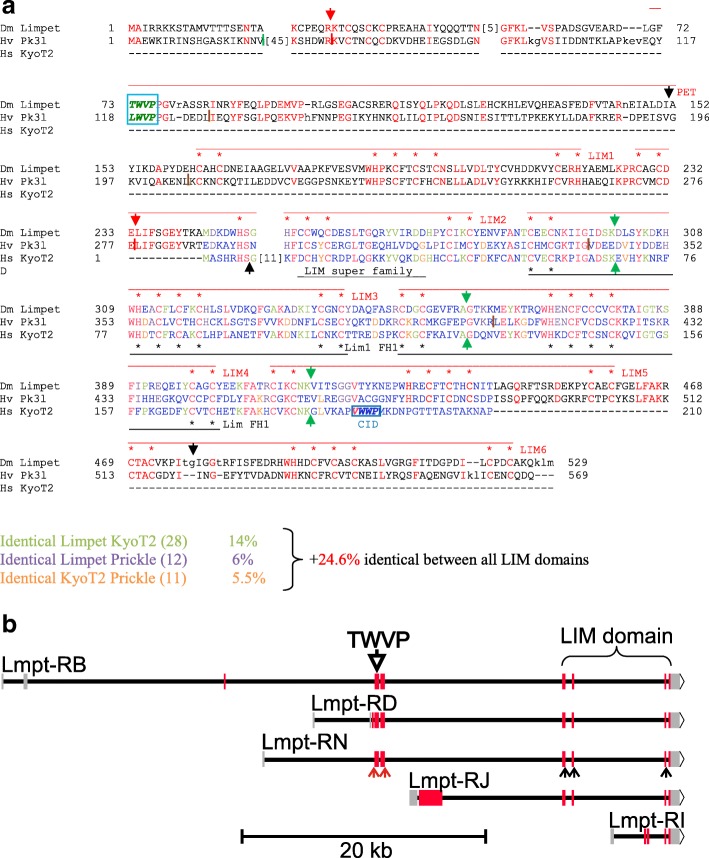
Alignment of fly Limpet, human KyoT2 and *Hydra* Prickle 3 like. **a** The alignment shows good conservation in the LIM domain between all three proteins. Best identity score in the LIM domain is between KyoT2 and Limpet (38.6%), followed by Limpet and Prickle 3 like (30.6%) and KyoT2 and Prickle 3 like (30.1%). Limpet and Prickle 3 like share considerable conservation over the entire length; the TWVP tetra-peptide motif is present at the identical position in the PET domain (italic green, boxed). Moreover, the two share conserved intron positions (red arrows and dashes). Green arrows indicate identical intron positions between *Limpet* and KyoT2. Black arrows and dashes mark intron positions specific to *Hydra* Prickle 3 like. Red residues are identical in all three proteins, green identical between Limpet and KyoT2, pink identical between Limpet and Prickle 3 like, and orange identical between KyoT2 and Prickle 3 like, blue are not conserved. **b** Scheme of the genomic organization of the *D. melanogaster Limpet* gene with representative transcripts (adapted from flybase). 15 transcripts are predicted, however, with 8 different open reading frames that fall into three classes: One encoding PET and LIM domain proteins (RB, RD and RN are shown as examples), one encoding LIM domain only proteins (RI, and RJ are shown as example), and the third containing neither (not shown). The exon encoding the PET domain with the TWVP tetra-peptide is indicated by the open arrow. The small downstream exons encode the LIM domain. Red arrows indicate exon/intron boundaries conserved between *Limpet* and Prickle 3 like, black arrows indicate exon/intron boundaries conserved between *Limpet* and KyoT2

**Fig. 10:**
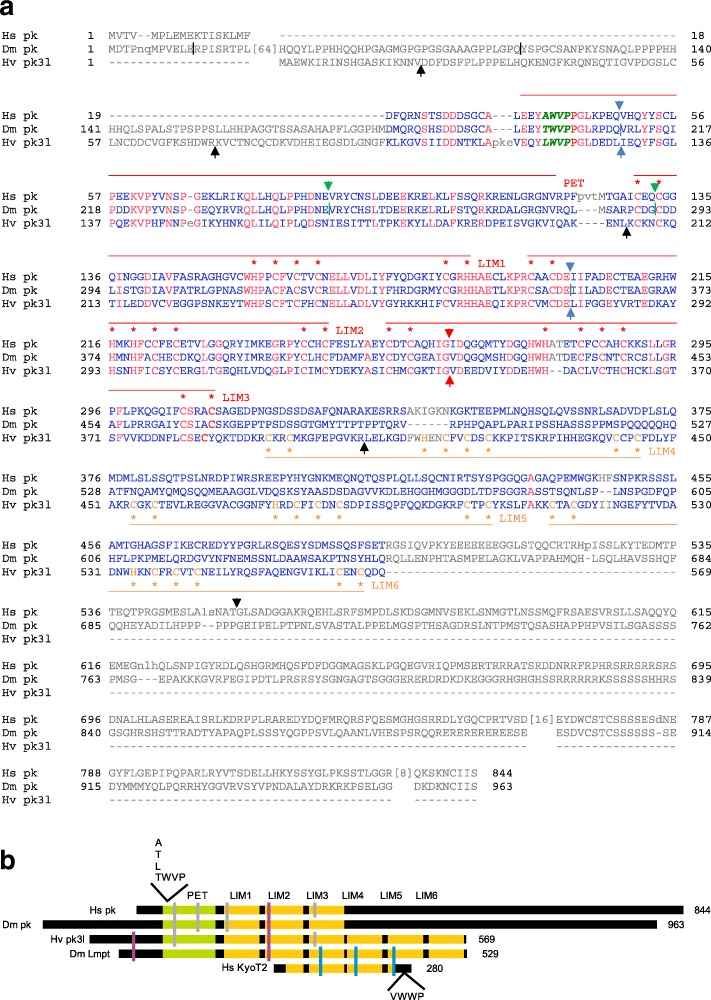
Conservation of Prickle like proteins. **a** Alignment of human Prickle homolog 2 (HS pk; GenBank AAI19003.1), *Drosophila melanogaster* Prickle isoform A (Dm pk; flybase) and *Hydra vulgaris* Prickle 3 like (Hv pk3l). All three share the PET domain and the first three LIM domains. In this part many residues are identical (red), blue residues are not conserved. The *Hydra* protein has three additional LIM domains, whereas the human and fly Prickle proteins extend C-terminally with no further similarities or discerned motifs. Reduced stringency conditions were used to allow alignment of LIM domain three. Notably, a tetra-peptide motif TMVP with high similarity to the Lmpt motif is shared by all Prickle proteins at the corresponding position (italic green) in the PET domain. Blue arrows indicate intron positions identical in all genes, green identical in human and *Drosophila* and red identical between human and *Hydra*. Black arrows and dashes present species specific introns. **b** Schematic comparison of Prickle (pk), Prickle 3 like (pk3l) and Limpet (Lmpt) proteins from human (Hs), *Drosophila* (Dm) and *Hydra* (Hv). PET and LIM domains are color coded green and yellow, respectively. Intron positions are indicated by dashes: purple conserved in *Limpet* and *prickle*, blue conserved between KyoT2 and *Drosophila Limpet* and grey conserved in *prickle*. Only conserved intron positions are shown. Positions of defined CID motif in KyoT2 (VWWP) and potential interacting motifs (TWVP like) in the others are indicated

Lmpt orthologs are specific to higher insects including the red flour beetle (*Tribolium castaneum)* (Fig. 8). The prediction for the *Tribolium Lmpt* gene is preliminary though, and differs from that of the EnsemblMetazoa database. The Lmpt-motif is always found in the PET domain of Lmpt homologues from any insect species analyzed. *Tribolium* Lmpt, however, is more diverged (TFVP instead of TWVP) (Fig. 8). Less stringent alignment conditions uncovered sequences similar to Lmpt in distant species, primarily based on the conservation of the PET and the LIM domains. Even *Hydra vulgaris* encodes a similar protein called Prickle 3 like that shares the overall structure with Lmpt, i.e. six Lim domains and a N-terminal PET domain with a motif similar to the Lmpt-motif, LWVP (Fig. 9a). The alignment reveals that this motif is at identical protein position compared to the one in Lmpt. Moreover, two introns of the *Hydra* Prickle 3 like gene, located outside of the KyoT2 homology, are at identical positions to the ones in the *D. melanogaster Lmpt* splice variant used here (Fig. 9a,b, Fig. 10b).

Low stringency database searches with either KyoT2 or Lmpt fetched homologues in all metazoan species including sponges and Placozoa, where they had been annotated as Prickle, Prickle like or Testin (Additional file 1 Dataset 1). Alignments of human and *Drosophila* Prickle with Hydra Prickle 3 like revealed a surprising similarity between the LIM domains of all these proteins (Fig. 10 a). Similar to Limpet but in contrast to KyoT2 they also share the PET domain with a presumptive Lmpt-motif plus additional LIM domains. Comparison of Prickle genes, including the ones from human, uncovered shared intron positions, reflecting common ancestry (Fig. 10 a, b). However, one must take into account that several splice variants for the three human Prickle genes are annotated. Moreover, *Hydra* Prickle 3 like clearly shares a stronger relationship with *Drosophila* Lmpt by the overall structure than with *Drosophila* or human Prickle (Fig. 10 b). The former contains six LIM domains, whereas Prickle homologues possess only three, and the third LIM domain does align just partly under standard condition. In fact, the *C. elegans* Prickle like protein contains only two LIM domains (GenBank: CCD62013.1 and CCD62014.1). Yet, conservation of intron position in the common alignment is striking: two are found conserved between all three homologues and three are conserved between two homologues, only two are specific to *Hydra*.

Notably, Prickle like proteins, here characterized by the shared PET and two to six LIM domains, all bear sequences similar to the Lmpt-motif at identical position at beginning of the PET domain that may represent a CSL binding motif (Fig. 10 a). This study hence predicts a novel CSL interactor present throughout the animal kingdom, provided the functionality of Lmpt-motif is confirmed experimentally in the future.

## Discussion

The goal of this work was an in silico analysis of potential repressors of CSL mediated Notch activity throughout the evolution of higher animals. The majority of experimental work so far addressed Notch repression mechanisms in vertebrates and in insects [64]. These experiments revealed two different principles of repression: the assembly of a repressor complex on Notch target gene promoters on the one hand, and the competition for CSL binding between activating NICD and repressor molecules on the other hand. Whereas the former mechanism is found in both, arthropods and vertebrates, direct competition for BTD binding appears to be restricted to vertebrates only.

### Repressor complex formation

CSL itself is central to repressor complex formation by recruitment of specific co-repressors with high affinity. The best experimentally characterized co-repressor is the Hairless protein of *Drosophila melanogaster*, where manifold genetic and molecular data on its functions have been gathered over the decades. Recently, the structure of the repressor complex was solved. *Drosophila* Hairless protein binds to the fly CSL ortholog Su(H) with the same affinity as NICD. Binding occurs at the CTD of Su(H) at places different from NICD, however, the resultant distortion of the CTD structure excludes NICD binding [33, 37]. Hence, binding of NICD and Hairless to Su(H) are mutually exclusive, resulting in an indirect competition of the two proteins. Due to the extremely high conservation of CSL proteins, fly Hairless can form complexes with mouse RBPJ, and can repress Notch activity in cell culture assays [37]. Hence, a conservation of Notch repression mediated by a Hairless-type protein was expected in vertebrates as well. However, the present analysis does not support this idea.

**Fig. 11:**
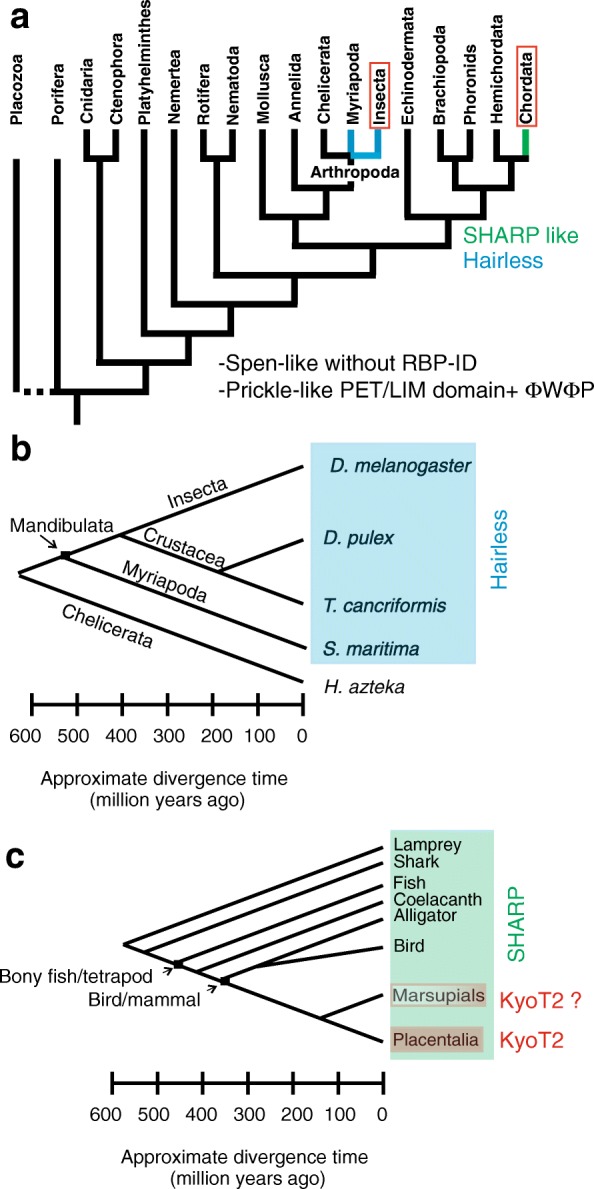
Phylogeny of CSL transcription complexes. **a** Phylogenetic tree of the various animal clades from this study. Notch repressors Hairless, SHARP, and KyoT2 were not found outside the clade, where they have been originally identified. The Spen-like and Prickle-like family of proteins, however, is present in all the studied metazoan animals. **b** Phylogeny of *Hairless* evolution. *Hairless* can be traced back to Myriapoda that diverged from dipteran flies approximately 500 million years ago, i.e. after the Mandibulata-Chelicerata radiation. **c** Phylogeny of SHARP/MINT and KyoT2 evolution. SPEN-like proteins containing a RBPJ interacting domain (i.e. SHARP) are only found in vertebrates starting with arctic lampreys as example for Cyclostomata. Lampreys are considered living fossils that may not have changed morphologically the past 500 million years, but diverged about 600 million years back from mammals. The KyoT2 co-repressor, however, is only found in placental mammals. The CSL interacting motif in marsupial KyoT2 is quite diverged, presumably impeding the binding to CSL

*Hairless* is a fast evolving gene [9, 54], however, *Hairless* orthologs can be tracked in all insect species analyzed to date. Moreover, the *Hairless* gene from the water flea *Daphnia pulex* is still biologically active in *Drosophila melanogaster*, despite its divergence [44]. This in silico analysis found evidence for a *Hairless* ortholog outside of Pancrustacea, in the Myriapoda *Strigamia maritima* (Fig. 11 a, b), which however, is highly diverged. Overall this study suggests the introduction of Hairless as a CSL co-repressor about 500 million years ago after the Mandibulata-Chelicerata radiation in the evolution of Panarthropoda [55, 65], since no *Hairless* ortholog in more distant species was found (Fig. 11b).

SHARP (or MINT) similarly assembles a repressor complex with CSL plus co-repressors, and is thus considered a functional homolog of Hairless [13]. Repressor activity was demonstrated experimentally in cell culture as well as in vivo in *Xenopus* embryos [48] and in a floxed mouse strain [66]. SHARP binds the mammalian CSL ortholog RBPJ (or CBF1) both at the BTD and at the CTD [49]. SHARP-BTD contacts imply competition with the Notch RAM domain [47–49]. SHARP can be therefore considered a dual repressor of Notch target genes by exerting both principal repression mechanisms, competition and repressor complex formation. SHARP belongs to the highly conserved group of SPEN proteins found throughout the animal kingdom that all share RRM and SPOC domains. The well-annotated genomes of fly, nematode and human contain each one copy of SPEN (respectively SHARP) and of NITO proteins [52]. However, the RBP-ID is specific to vertebrate SHARP proteins down to arctic lamprey (Fig. 11 c), and is extremely well conserved. The conservation extends beyond the determined interaction domain, suggesting additional as yet uncharacterized functions. The RBP-ID, however, is not found in lower chordates like Amphioxus (lancelets) (Additional file 1 Dataset 1), suggesting its introduction into a SPEN precursor about 600 million years ago during chordate evolution [67].

### Competitors of Notch

Upon ligand-receptor contact Notch is cleaved at the membrane, and the intracellular domain NICD is released acting as a co-activator of CSL. NICD makes twofold CSL contacts, first with the RAM domain at the BTD, and second with the ANK repeats at the CTD [11–14]. In *C. elegans*, RAM binding precedes and facilitates ANK contacts, whereas in *Drosophila* NICD-Su(H) contacts are stable in the absence of RAM [9, 11, 68]. Overall, the RAM domain of Notch is not well conserved. Notch proteins from man to worm share basic residues at the N-terminus of RAM, and a tetra-peptide ΦWΦP motif that binds a non-polar pocket of the BTD with high affinity [11–14, 23]. In *Hydra vulgaris* Notch, however the ΦWΦP motif is altered to LWKF (Fig.1c). Mutation of the Proline residue to Alanine inhibits NICD binding by more than 300 fold [23]. Hence, the RAM-BTD interaction in *Hydra* is presumably very weak if not stabilized otherwise. Perhaps, the high affinity RAM-BTD interaction has been gained in the course of evolution in some clades, explaining the differential requirement in flies versus worm or mammals. Moreover, the high affinity binding may have started repressor evolution as further means of regulation.

The hydrophobic pocket of the BTD is targeted by a number of proteins that compete with NICD for the binding of CSL. These include the viral trans-activator EBNA2 as well as the repressors RITA and KyoT2. EBNA2 hijacks the Notch signaling pathway, stimulating cell proliferation by the activation of Notch target genes [22]. RITA not only competes with NICD, but also causes a cytoplasmic translocation and retention of RBPJ, thereby reducing its availability for activated Notch [26]. KyoT2 has been shown in reporter assays to compete with both, NICD and EBNA2 for the binding of RBPJ, thereby inhibiting transcription of Notch reporter genes [21]. The EBNA2 contact site was determined by mutational analyses of CSL [69, 70]. Detailed experimental and structural insights are available for RITA and KyoT2 binding of CSL, revealing striking similarities between the structures of the BTD-RAM and the BTD-repressor complexes [24, 25]. All these proteins share the ΦWΦP motif that contacts the BTD (Fig. 1 c) apart from further amino acids that specify the interaction [24, 25]. SHARP may make similar BTD contacts despite the lack of a typical ΦWΦP motif [49].

This work concentrated on the evolution of KyoT2, a specific splice form of KyoT, which harbors a CSL interacting domain (CID) specified by the ΦWΦP tetra-peptide motif. These analyses reveal the presence of KyoT2 homologues including CID only in placental mammals, and even not in marsupials, indicating the introduction of the CID roughly 100 million years ago. KyoT2 contains two and a half LIM domains that share highest homology with *Drosophila* Lmpt. The relationship of the two genes is strongly supported by conserved intron/exon boundaries. Lmpt shares good similarity with *Hydra* Prickle 3 like: both not only contain six LIM domains but also a PET domain. This classifies them as members of the Prickle protein family, which apart from PET generally contain 2–3 LIM domains. PET is an acronym for the *Drosophila* proteins **P**rickle, **E**spinas and **T**estin, and respective homologues are present throughout the animal kingdom (see Additional file 1 Dataset 1). Interestingly, the well-conserved PET domain harbors a ΦWΦP motif at its N-terminus, which may have the potential to bind the BTD of CSL proteins. The human genome encodes three Prickle proteins in several splice variants, and two proteins more closely related to *Drosophila* Testin. All three human Prickle genes harbor a PET domain with the respective ΦWΦP tetra-peptide motif at the N-terminus. In Testin proteins in contrast, the first residue of the corresponding four amino acids is highly charged. Experimental evidence for respective interactions with CSL, however, is lacking. *Drosophila* Lmpt, for example, has been involved in the defense response to fungal and bacterial infections [62]. Moreover, downregulation of Lmpt activity resulted in an enhancement of phenotypes induced by a mutant form of the human androgen receptor in the fly. In these experiments, *Drosophila* was used as model organism to study candidate genes known from human to be involved in spinobulbar muscular atrophy [71]. Prickle function has been studied in greatest detail in *D. melanogaster*, as it is pivotal to planar cell polarity (PCP). Prickle belongs to the intracellular core PCP factors that stabilize PCP complexes and orient cells relative to Wnt/Wingless signals within a tissue, not only in *Drosophila* but also in man (reviewed in: [72–74]). Despite the fact that interactions between Wingless and Notch signaling pathway are well documented (reviewed in: [75, 76]), a direct link between Notch activity and Prickle remains to be established. If it were confirmed, this work has uncovered a novel, very ancestral CSL interactor found in the entire animal kingdom including Protostomia and Deuterostomia.

## Conclusion

Mechanisms of Notch repression appear to be specific to either chordates or higher arthropods. Orthologues of experimentally validated repressors were not found outside the phylogenetic group they have been originally identified. However, the data provide a hypothesis on the evolution of mammalian KyoT2 repressor from Prickle like ancestors. Moreover, the finding of a potential CSL interacting motif in Prickle homologues opens the possibility of a novel Notch repressor system present throughout the whole animal kingdom.

Only in chordates and insects Notch repressors are experimentally characterized. Even in nematodes represented by *C. elegans* no repressors have been identified so far, despite the extremely well experimentally based study of the Notch signaling pathway. This study found an intriguing similarity of the LIM domain proteins murine KyoT2, *Drosophila* Limpet and *Hydra* Prickle 3 like, that all contain a novel, putative CSL interacting motif. Future work may establish Prickle like proteins as novel, very ancestral CSL interactors with an important role in Notch repression. However, only experimental studies can prove or disprove this hypothesis.

## Material and methods

### Database searches

The following databases were used to identify genes in different species: basic local alignment search tool of NCBI (https://blast.ncbi.nlm.nih.gov/Blast.cgi), Ensembl (http://www.ensembl.org/index.html; http://metazoa.ensembl.org/index.html) and flybase (http://flybase.org) using tblastn as search tool for similar protein sequences. Alignments were done against nucleotide collections, whole genomic DNA, whole genome shotgun contigs, expressed sequence tags (est) and cDNA under standard conditions. If no similarity could be found, the search was redone under less stringent conditions.

### Sequence analyses

Most analyses have been done with the embnet HUSAR database (https://genome.dkfz-heidelberg.de) using the following programs: restriction site and translation in all six frames, dotplot, multiple alignment (prrn), and several protein analyses programs. Unfortunately the data base closed service by December 2017, therefore, further analyses were done used ncbi services: conserved domain search (CD search), and COBALT for multiple alignments. Additional dotplot analyses was performed with EMBOSS explorer (http://www.bioinformatics.nl/cgi-bin/emboss/dottup). Possible nuclear export signals were searched with the NetNES 1.1 Server (http://www.cbs.dtu.dk/services/NetNES/) [45].

### Gene annotations

Gene annotations were done manually. Therefore, the genomic sequence was translated in all three frames and then scanned for exon/intron boundaries (gt/ag rule), which maintain the open reading frame over introns. Afterwards the sequence was compared with sequences of prospective protein homologues and evaluated for improvements by sequence similarities.

### Protein structure predictions

Protein structures of protein orthologs were predicted by alignment with known structures using SWISS Model (https://swissmodel.expasy.org) [77] and further analyzed by MacPyMOL.

## Additional Files

Additional file 1: Dataset 1 with Gene accession numbers and sequences retrieved from database or predicted from whole genome sequences used for comparisons in this work; (PDF 230 kb)

Additional file 2: **Figure S1** with Structure prediction of the *StmaH* gene. (PDF 47 kb)

Additional file 3: Dataset 2 with List of species including their common names used for the alignments of the respective gene; (PDF 121 kb)

## Abbreviations

ANK: Ankyrin repeats
BTD: **b**eta **t**refoil **d**omain (in CSL)
CBD: **C**tBP **b**inding **d**omain (in H)
CBF1: **C**-promoter **b**inding **f**actor 1
CID: **C**SL **i**nteracting **d**omain
CSL: Acronym of **C**BF1, **S**u(H), **L**ag-1
CT box: **C**-**t**erminal part of SBD
CtBP: **C**-**t**erminal **b**inding **p**rotein
CTD: **C**-**t**erminal **d**omain (in CSL)
CtIP: **C**tBP **i**nteracting **p**rotein
DmH: *Drosophila melanogaster Hairless* gene
EBNA2: **E**pstein-**B**arr **n**uclear **a**ntigen **2**
Fhl1: **F**our and a **h**alf **L**im domains 1
GBD: **G**ro **b**inding domain (in H)
glp-1: Anormal **g**erm **l**ine **p**roliferation 1
Gro: Groucho
H: Hairless
HES: **H**airy-**E**nhancer of **s**plit
Isl1: Islet-1 insulin gene-enhancer-binding protein
lag 1: **L**in-12 **a**nd **G**lp-1 phenotype
LIM: Acronym of **L**in-11, **I**sl1, **M**EC-3
lin-12: Abnormal cell **lin**eage 12
Lmpt: Limpet
Mam: Mastermind
MAML: Mastermind like
mec-3: **Mec**hanosensory abnormality
MINT (or SHARP) **M**SX2: **I**nteracting **n**uclear **t**arget protein
MSX2: Muscle segment Homeobox protein-2
N: Notch
NES: **N**uclear **e**xport **s**ignal
NICD: **N**otch **i**ntra**c**ellular **d**omain
Nito: Spenito
NLS: **N**uclear **l**ocalization **s**ignal
NT box: **N**-**t**erminal part of SBD
NTD: **N**-**t**erminal **d**omain (in CSL)
PCP: **P**lanar **c**ell **p**olarity
PET: Acronym of **P**rickle, **E**spinas, **T**estin
Pk: Prickle
RAM **R**BP-Jκ: **a**ssociated **m**olecule
RBP-ID **RBP**J: **i**nteracting **d**omain
RBPJ: **R**ecombination signal sequence-**b**inding **p**rotein **J**κ
RID: **R**eceptor **i**nteracting **d**omain
RITA **R**BP-J: **I**nteracting and **t**ubulin **a**ssociated
RRM RNA: Recognition motif
SBD: **S**u(H) **b**inding **d**omain (in H)
SHARP (or MINT) **S**MRT/**H**DAC1: **A**ssociated **r**epressor **p**rotein
Spen: Split ends
SPOC: Spen paralog and ortholog C-terminal
Su(H): Suppressor of Hairless
Φ,: Any hydrophobic amino acid
