# Supplementary material for: The evolution of transcriptional repressors in the Notch signaling pathway: a computational analysis
Source: Hereditas. 2019 Jan 17;156:5. doi: 10.1186/s41065-019-0081-0 (PMC6337844; doi:10.1186/s41065-019-0081-0)
Supplement: Supplementary file 1 — Dataset 1 with Gene accession numbers and sequences retrieved from database or predicted from whole genome sequences used for comparisons in this work; (PDF 230 kb) [file 41065_2019_81_MOESM1_ESM.pdf]

The evolution of transcriptional repressors in the Notch signaling pathway throughout the animal kingdom: a computational analysis

Dieter Maier

Additional Dataset 1

Gene accession numbers and sequences retrieved from database or predicted from whole genome sequences used for comparisons in this work.

Suppressor of Hairless Su(H)

Hydra vulgaris

Su(H) GenBank: CDG70345.1

Triops cancriformis

Triops cancriformis DNA, contig: contig_1875-19264, whole genome shotgun sequence; Sequence ID: BAYF01001879.1, Length: 19264

```
MFCSEAWRVACRWLVWLGSWKPPQQYTIVYVRLALAERSKSPALYVVLQPPPETIPCSQMEV
WEPHRGPPLFRPKEQRLTREAMMRYLQERGDMVLVILHAKVAQKSYGNEKRFFCPPPCIYLY
GDGWRRRREQLQREATAAGASAAEAEASSQLCAFIGIGNSDQDMQQLDLNGKHYCAAKTLFI
SDSDKRKHFMLSVKMFYGNGQDIGVFHSKRIKVISKPSKKKQSLKNADLCIASGTRVALFNR
LRSQTVSTRYLHVENNNFHASSTQWGAFTIHLLDDSESESEEFTVRDGYIHYGSTVKLVCSV
TGMALPRLIIRKVDKQMAVLDADDPVSQLHKCAFYMKDTERMYLCLSQERIIQFQATPCPKE
PNKEMINDGAAWTIISTDKAEYQFFEGMGPVRSPVTPVPIVHSLHLNGGGEVAMLELTGENL
TPNLKVWFGDVEAETMFRCEESMLCVVPDISAFRSGWQWVRHPTQVPVSLVRSDGVIYATRL
TFTYTPEPGPRPHCPTTDEVLRGSVSTNGSSHMVGGNMHYEHLPQGPTM (545)
```

Strigamia maritima

Strigamia maritima strain Brora ctg7180001225943, whole genome shotgun sequence. Sequence ID: AFFK01015431.1, Length: 31215

```
MNEVFLPQGQYDYPPPLASTYSREADLWNVNLATYSSAPTTCTGATPAPSVTGFYAQATGSNSVSPSSVSLTTLT
PHFADNHPVDLSNSHRGEGGHLDLVRFQSDRVDAYKHANGLSVHIPDHHDATSHMIAGSLTPPDKVNGEHGHQLV
TMSNASQMSLGSIASSLQTPPSPIPTPSPPVPLDGVRHSHKDQRLTREAMKRYLRERGDQVLVILHAKVAQKSYG
NEKRFFCPPPCIYLFGDGWRRKREQMLHEGETEQGAQLCAFIGIGNSDQDMQQLDLNGKNYCAAKTLYISDSDKR
KHFMLTVKMFYGNGEDIGVFHSKRIKVISKPSKKKQSLKNADLCIASGTKVALFNRLRSQTVSTRYLHVENGTFH
ASSTQWGAFTIHLLDDNESESEEFTVRDGYIHYGSTVKLVCSVTGMALPRLIIRKVDKQTALLDADDPVSQLHKC
AFYMKDTERMYLCLSQERIIQFQATPCPKEPNKEMINDGASWTIISTDKAEYTFCEGMGPVRTLVTPVPVVHSLH
LNGGGDVAMLELTGENFTPTLRVWFGDVEAETMYRCQESMLCVVPDISAFRGGWQFVRQPTQVPVSLVRSDGIIY
ATGLTFTYTPEPGPRPHCPA TDHILRGGSQSGLDRLPTSPDPALTYNPPHHALTPI
```

Hairless H

Triops cancriformis

Predicted from whole genome shotgun sequence Contig_1325-52010 (BAYF01001327.1)
MKCCEPPHSQLIMSEEQDIEAGLMKPKIEEPLLVQSTAPIVSPTLAKHSEVENNGSCSPFTP
EPVAEPKPNGSRLKFFFDGKMVLELSDKKDGEKVSWVPVTKKTYWPPPLPGRPLRQESTTSY
SGNASFSTDQGSYLSDSCLSKSCSLVSDDSSVQSSPWQRDTRWKQPAPKRARCQDRTFYMLV
PKSERSRRCSSHVQYRKPFLVQLPHCQCKRTTTRPRGAEPTKRRLSLPRMVQKLGERASAMS
RAAEFSTHSVRIKEEWRPCEIRSAKDFHLTAVLGSPEQSTRKRAWKESDRARSADAYLKKTK
AQQPVSALVAAPLALSSNSKGFDKHSSYSIDSLLAKDSLNSASANPEVNHGPSFLRTLLKAP
ASSVSPASGSSPSSAAVTLANKVKVNSASPSRNGHSKGDSASTPRHSPRSQVPTPPQDAAGL
QLPRTSGGGTPRPSYSASPGPRFNPAVLTGLLPRMAGIPGLPPPWEMYNPYMTQLLGASPSW
MSYSWPPPLGALPSAAFGFSEGLMMMPPNAAPSAIGHKTNGASIAESDNCSSSSSSSNSRSV
IVKPSSTSKESRKSVEDALIQVPLNLSKSRSGESR

Strigamia maritima

SMARO13018 from flybase
MLNSFSISSRGLSRAKMPNPNCETCESSQNNKRKCENNANVQSGNKKRSTDPNECQQPAQSA
HDFLKKLVHEQKLHAPTLFNNTNELKPENNRTGSSENDTNGKLSFYLGENGRFILELEHKPG
GKRRNGGWIQTKGITWMPRPEEEKLINPIKQELKESETSRMKLKNLKRFSSQRETTNACSVV
LFNFRINRRRDFKHFPRLNAYYLRKAARHPFSIDLFFFSKSEKCKTAPPSTKLVKKRTKRLN
DLFRPPEFVNSTADNVKRWFDEVGSVKVKCEPDNASLPLPEWYLDPSKSQIPVNEMIGNPRP
YLPAAPTHYDDKESNVNSMSEPLDLRTNVVRETKRDEWMKREAKEHPGNAFYVGLIPFPLPG
HGEYSAPSAVQPWPCVSCPVVAYAPTVVNSYYYCCCFLPGCRSNCLCKTEGKQLTSRSSEIP
KECDCATDLTNACNKKFPIPVAVKSDHQTPGKKRMSETDVRIQC

Predicted from whole genome shotgun sequence Contig_ 7180001234497; (AFFK01023985.1)
MLNSFSISSRGLSRAKMPNPNCETCESSQNNKRKCENNANVQSGNKKRSTDPNECQQPAQSA
HDFLKKLVHEQKLHAPTLFNNTNELKPENNRTGSSENDTNGKLSFYLDGRFILELEHKPGGK
RRNGGWIQTKGITWMPRPEEEKLINPIKQELKESETSRMKLKNLKRFSSQRETTNACSVVLF
NFRINRRRDFKHFPRLNAYYLRKAARHPFSIDLFFFSKSALEDDHDGEQQDSLADKQRHQLK
HRELFLSRQVETLPATHIRGKCTVTLLNETESLLSYLNKDVNLNDFYMEFKCKICFFLCFFR
THFFIRWFTIPNKRHYWLIKEKSVSVQVTFQFISYFENLLNEAAMLNPLTAAEAIARDRDVS
VDFFDFLADHVAVQRDFRCEEKLFLHEKVHFAGRVRFHRHRNVSFALQVAAQFLQPVASLTG
PLNLRQQFLEIKNK

Notch:

Hydra vulgaris

NotchGenBank: ABV68547.1

SHARP/MINT

Homo sapiens (Human)

msx2-interacting protein
NCBI Reference Sequence: NP_055816.2

Bos taurus (Cattle)

TPA: spen homolog, transcriptional regulator, GenBank: DAA21211.1

Lipotes vexillifer (chinese dolphin)

SPEN (Protein ID XP_007459340.1)

Manis javanica (Sunda pangolin)

spen family transcriptional repressor (SPEN), transcript variant X1, mRNA
XP_017521424.1

Peromyscus maniculatus bairdii (Deer mouse)

spen family transcriptional repressor (Spen), transcript variant X2, mRNA
XP_015849836.1

Elephantulus edwardii (Cape elephant shrew)

spen family transcriptional repressor (SPEN), mRNA XP_006883683.1

Erinaceus europaeus (European hedgegog)

spen family transcriptional repressor (SPEN), mRNA XP_016043917.1

Alligator mississippiensis

spen family transcriptional repressor (SPEN), transcript variant X4, mRNA
XP_014453442.1

Zonotrichia albicollis (White throated sparrow)

spen family transcriptional repressor (SPEN), partial mRNA XP_014129084.1

Parus major (Great tit)

spen family transcriptional repressor (SPEN), transcript variant X2, mRNA
XP_015503523.1

SHARP/MINT (continued)

Haliaeetus leucocephalus (Bald eagle)

spen family transcriptional repressor (SPEN), transcript variant X1, mRNA
XP_010575514.1

Danio rerio (Zebrafish)

spen family transcriptional repressor (spen), transcript variant X2, mRNA
XP_009295192.1

Rhincodon typus (Whale shark)

msx2-interacting protein-like (LOC109926950), partial mRNA XP_020382431.1

Callorhinchus milii (Austalien ghostshark)

spen family transcriptional repressor (spen), mRNA XP_007905153.1

Latimeria chalumnae (West Indian Ocean coelacanth)

spen family transcriptional repressor (SPEN), transcript variant X1, mRNA
XP_005996344.1

Manis javanica (Sunda pangolin)

PREDICTED*: msx2-interacting protein isoform X1*
NCBI Reference Sequence: XP_017521424.1

Gadus morhua (Atlantic cod)

WGS project CAEA02000000 data, contig: gadMor2_ctg_224, whole genome shotgun sequence
Sequence ID: CAEA020000225.1,Length: 169981

Oncorhynchus kisutch (coho salmon)

isolate 150728-3 scaffold02541, whole genome shotgun sequence
Sequence ID: MPKV01002541.1,Length: 3463492,Number of Matches: 15

Leucoraja erinacea (Little skate)

LER_WGS_1_CONTIG_2664153, whole genome shotgun sequence
Sequence ID: AESE012501079.1,Length: 9851 **Not complete.**

SHARP/MINT (continued)

Crotalus horridus (Timber rattlesnake)

isolate 016-059-111 Sequence_21106_23757, whole genome shotgun sequence
Sequence ID: LVCR01021106.1,Length: 23757 Number of Matches: 10

Lethenteron camtschaticum (Arctic lamprey)
(sequence prediction made with five shotgun sequences)
contig033099, whole genome shotgun sequence
Sequence ID: APJL01092535.1, **Length: 6973**
contig051046, whole genome shotgun sequence
Sequence ID: APJL01092534.1, **Length: 4466**
contig124466, whole genome shotgun sequence
Sequence ID: APJL01137493.1, **Length: 1523**
contig021404, whole genome shotgun sequence
Sequence ID: APJL01091189.1, **Length: 10023**
contig168045, whole genome shotgun sequence
Sequence ID: APJL01173146.1, **Length: 1055**

KyoT2:

Lipotes vexillifer (Chinese river dolphin)

PREDICTED: **four and a half LIM domains 1 (FHL1), transcript variant X6**, mRNA
Sequence ID: XM_007462905.1 Length: 633 Number of Matches: 1

Phascolarctos cinereus (koala)

PREDICTED: **four and a half LIM domains 1 (FHL1), transcript variant X3**, mRNA
Sequence ID: XM_020969789.1 Length: 611 Number of Matches: 2

Monodelphis domestica (Gray short-tailed opossum)

PREDICTED: **four and a half LIM domains 1 (FHL1), transcript variant X1**, mRNA
Sequence ID: XM_001379142.4 Length: 2324 Number of Matches: 5

Danio rerio (Zebrafish)

four and a half LIM domains 1a (fhl1a), mRNA
Sequence ID: NM_001007287.1 Length: 1435 Number of Matches: 5

Salmo salar (Atlantik salmon)

PREDICTED: **Four and a half LIM domains protein 1 (fhl1), transcript variant X1**, mRNA; Sequence ID: XM_014199413.1 Length: 1496 Number of Matches: 5

Clupea harengus (Atlantik herring)

PREDICTED: **four and a half LIM domains 1 (fhl1)**, mRNA
Sequence ID: XM_012841176.1 Length: 921 Number of Matches: 5

KyoT2 (continued):

Alligator mississippiensis

PREDICTED: **four and a half LIM domains 1 (FHL1), transcript variant X1**, mRNA
Sequence ID: XM_019492797.1 Length: 1577 Number of Matches: 3

Haliaeetus leucocephalus (Bald eagle)

PREDICTED: **four and a half LIM domains 1 (FHL1), transcript variant X2**, mRNA
Sequence ID: XM_010572470.1 Length: 980 Number of Matches: 5

Parus major (Great tit)

PREDICTED: **four and a half LIM domains 1 (FHL1), transcript variant X4**, mRNA
Sequence ID: XM_015626478. 2Length: 1857 Number of Matches: 4

Felis catus (Cat)

PREDICTED: **four and a half LIM domains 1 (FHL1), transcript variant X6**, mRNA
Sequence ID: XM_019824360.1 Length: 2163 Number of Matches: 3 Cat

Canis lupus (Gray wolf) familiaris

PREDICTED: **four and a half LIM domains 1 (FHL1), transcript variant X5**, mRNA
Sequence ID: XM_861215.5 Length: 2380 Number of Matches: 5 Dog

Monodelphis domestica (Gray short-tailed opossum)

PREDICTED: **four and a half LIM domains 1 (FHL1), transcript variant X1**, mRNA
Sequence ID: XM_001379142.4 Length: 2324 Number of Matches: 5

Limpet:

Drosophila melanogaster

Limpet Lmpt, ID: FBgn0261565 **Symbol**: Lmpt-PN

Musca domestica (Housefly)

PREDICTED: **four and a half LIM domains protein 2** (LOC101894619), transcript variant X7, mRNA
Sequence ID: XM_005190021.3 Length: 2184 Number of Matches: 1

Anopheles gambiae (mosquito)

str. PEST **AGAP005400-PA** (AgaP_AGAP005400) mRNA, complete cds
Sequence ID: XM_315409.4 Length: 2251 Number of Matches: 5

str. PEST **AGAP005398-PA** (AgaP_AGAP005398) mRNA, complete cds
Sequence ID: XM_001237655.2Length: 933Number of Matches: 4

Aedes aegypti (mosquito)

PREDICTED**: four and a half LIM domains protein 2** (LOC5573016), transcript variant X1, mRNA
Sequence ID: XM_021843544.1 Length: 3474 Number of Matches: 1

Tribolium castaneum (red flour beetle)

EnsemblMetazoa database predicts **two different genes** TC013758_001 (encoding PET and one LIM domain) and TC013756_002 (encoding three LIM domains) in close neighborhood.

PREDICTED: **four and a half LIM domains protein 2** (LOC661300), transcript variant X3, mRNA
Sequence ID: XM_970928.4 Length: 2703 Number of Matches: 1
This prediction in NCBI corresponds my own annotation

Apis dorsata (Giant honey bee)

PREDICTED: **four and a half LIM domains protein 3-like** (LOC102677765), transcript variant X2, mRNA
Sequence ID: XM_006621240.1Length: 2209Number of Matches: 1

Prickle (alignments):

Hydra vulgaris (fresh water polyp)

strain 105 ctg1101284810209, whole genome shotgun sequence
Sequence ID: ABRM01028485.1 Length: 11387 Number of Matches: 5

PREDICTED: **Hydra vulgaris prickle-like protein 3** (LOC100212931), transcript variant X2, mRNA
Sequence ID: XM_002158307.3 Length: 2181 Number of Matches: 1

Homo sapiens (Human)

prickle planar cell polarity protein 2 (PRICKLE2), mRNA
Sequence ID: NM_198859.3 Length: 8322 Number of Matches: 1

Drosophila melanogaster

Prickle Pk, ID: FBpp0088113 **Symbol:** pk-PA

Prickle (phylogeny):

Chordata: *Homo sapiens* (man)
PRICKLE2, mRNA, Sequence ID: NM_198859.3

Hemichordata: *Saccoglossus kowalevskii (acorn worm)*
prickle2-like protein mRNA (partial) Sequence ID: GU224218.1

Phoronids: *Phoronis australis* (horseshoe worm)
whole genome shotgun sequence; Sequence ID: NMRA01000005.1

Brachiopoda: *Lingula anatina*
prickle planar cell polarity protein 3-A transcript variant X3, mRNA, Sequence ID: XM_013549311.1

Echinodermata: *Strongylocentrotus purpuratus* (purple sea urchin)
prickle-like protein 3 transcript variant X4, mRNA, Sequence ID: XM_011685458.1

Insecta:*Drosophila melanogaster* (vinegar fly)
Prickle Pk, Sequence ID**:** FBpp0088113

Myriapoda: *Strigamia maritima*
whole genome shotgun sequence, Sequence ID: AFFK01019870.1

Prickle (phylogeny) continued:

Chelicarata: *Centruroides sculpturatus* (bark scorpion)
prickle planar cell polarity protein 3-like, transcript variant X3, mRNA, Sequence ID: XM_023383390.1

Annelida: *Helobdella robusta* (leech)
hypothetical protein partial mRNA, Sequence ID: XM_009015555.1

Nematoda: *Caenorhabditis elegans* (nematode worm)
Prkl-1 coding sequence, Sequence ID: NM_171372.2

Mollusca: *Lottia gigantean* (owl limpet)
hypothetical protein partial mRNA, Sequence ID: XM_009055231.1

Rotifera*: Brachionus plicatilis* (wheel animal)
whole genome shotgun sequence, Sequence ID: REGN01008705.1

Nemertea: *Notospermus geniculatus* (ribbon worm)
whole genome shotgun sequence, Sequence ID: NMRB01001797.1

Platyhelminthes: *Schistosoma mansoni* (blood fluke)
LIMPET mRNA, complete cds; Sequence ID: EU436753.1

Ctenophera: *Mnemiopsis leidyi* (warty comb jelly)
LIM protein mRNA, complete cds, Sequence ID: JN615190.1

Porifera: *Amphimedon queenslandica* (sponge)
testin-like, mRNA, Sequence ID: XM_003387784.2
testin-like, transcript variant X1, mRNA, Sequence ID: XM_011406892.2
testin-like, transcript variant X2, mRNA Sequence ID: XM_019998858.1

Placozoans: *Trichoplax adhaerens*
hypothetical protein, partial mRNA, Sequence ID: XM_002112366.1
