# Supplementary material for: The evolution of transcriptional repressors in the Notch signaling pathway: a computational analysis
Source: Hereditas. 2019 Jan 17;156:5. doi: 10.1186/s41065-019-0081-0 (PMC6337844; doi:10.1186/s41065-019-0081-0)
Supplement: Supplementary file 2 — Figure S1 with Structure prediction of the StmaH gene. (PDF 47 kb) [file 41065_2019_81_MOESM2_ESM.pdf]

**The evolution of transcriptional repressors in the Notch signaling pathway throughout the animal kingdom: a computational analysis**

Dieter Maier

## Additional Figure S1 **Structure prediction of the *StmaH* gene**

a

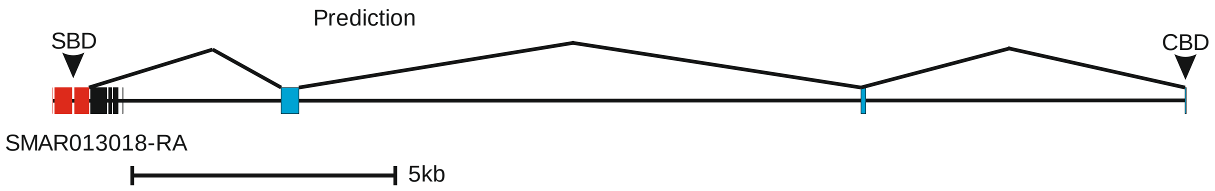


b

```
         GTTTTCACCGCCATCGAAATGTCTCGTTCGCTTTGCAAGTTGCCGCTCAATTCCTGCAAC
   23341 ---------+---------+---------+---------+---------+---------+ 23400
         CAAAAGTGGCGGTAGCTTTACAGAGCAAGCGAAACGTTCAACGGCGAGTTAAGGACGTTG

a        V  F  T  A  I  E  M  S  R  S  L  C  K  L  P  L  N  S  C  N   -
b         F  S  P  P  S  K  C  L  V  R  F  A  S  C  R  S  I  P  A  T  -
c          F  H  R  H  R  N  V  S  F  A  L  Q  V  A  A  Q  F  L  Q  P -

         CGGTTGCATCGCTGACCGGTCCGCTGAACTTGCGCCAGCAATTCCTAGAAATAAAAAATA
   23401 ---------+---------+---------+---------+---------+---------+ 23460
         GCCAACGTAGCGACTGGCCAGGCGACTTGAACGCGGTCGTTAAGGATCTTTATTTTTTAT

a        R  L  H  R  *  P  V  R  *  T  C  A  S  N  S  *  K  *  K  I   -
b         G  C  I  A  D  R  S  A  E  L  A  P  A  I  P  R  N  K  K  *  -
c          V  A  S  L  T  G  P  L  N  L  R  Q  Q  F  L  E  I  K  N  K -

         AAAAACAAAAAAAAACAAATAAAAATCAAAATAAAAATCACTTTTTATCACTTTACTTTG
   23461 ---------+---------+---------+---------+---------+---------+ 23520
         TTTTTGTTTTTTTTTGTTTATTTTTAGTTTTATTTTTAGTGAAAAATAGTGAAATGAAAC

a        K  N  K  K  K  Q  I  K  I  K  I  K  I  T  F  Y  H  F  T  L   -
b         K  T  K  K  N  K  *  K  S  K  *  K  S  L  F  I  T  L  L  C  -
c          K  Q  K  K  T  N  K  N  Q  N  K  N  H  F  L  S  L  Y  F  A -
```

a) Structure prediction for the *StmaH* locus based on sequence analysis of contig 7180001234497 of the whole genome shotgun sequence (ID: AFFK01023985.1, length 39743) for similarities. Red and black boxes depict exons predicted by OrthoDB (flybase), encoding the SMARO123018-TA protein with the potential Su(H) binding domain (SBD, arrowhead). According to my prediction, *StmaH* contains the red plus the blue exons, skipping the black ones. The prediction is based on open reading frame encoding a highly basic protein with potential NLS and derived GBD, taking GT/AG rule for exon/intron boundaries into account. The last predicted exon is roughly 22 kb downstream; it contains a well conserved CtBP binding motif (CBD), however, no suitable stop codon.

b) 3' genomic sequence of the last predicted *StmaH* exon. In red the predicted open reading frame ending with a potential CtBP interacting motif (highlighted in red). The sequence downstream is extremely AT rich, which is a sign of non-coding sequences. Stop codons are present in reading frames a and b (marked in blue), or could occur 2-3 codons downstream of the CBD in case of C/T misreading.
