# Supplementary material for: The evolution of transcriptional repressors in the Notch signaling pathway: a computational analysis
Source: Hereditas. 2019 Jan 17;156:5. doi: 10.1186/s41065-019-0081-0 (PMC6337844; doi:10.1186/s41065-019-0081-0)
Supplement: Supplementary file 3 — Dataset 2 with List of species including their common names used for the alignments of the respective gene; (PDF 121 kb) [file 41065_2019_81_MOESM3_ESM.pdf]

**The evolution of transcriptional repressors in the Notch signaling pathway throughout the animal kingdom: a computational analysis**

Dieter Maier

## Additional Dataset 2

**List of species including their common names used for the alignments of the respective gene:**

**Hairless:**

DapuH *Daphnia pulex* (water flea)

TrcaH *Triops cancriformis* (Tadpole shrimp)

StmaH *Strigamia maritima* (European centipede)

**SHARP:**

Hs *Homo sapiens* (human)

Bt *Bos taurus* (cattle)

Lv *Lipotes vexillifer* (Chinese river dolphin)

Pman *Peromyscus maniculatus* (deer mouse)

Eed *Elephantulus edwadii* (Cape elephant shrew)

Eeu *Erinaceus europaeus* (European hedgehog)

Mj *Manis javanica* (Malayan pangolin)

Am *Alligator mississippiensis* (American alligator)

Za *Zonotrichia albicollis* (sparrow)

Pmaj *Parus major* (great tit)

**SHARP (continued):**

Hl *Haliaeetus leucocephalus* (bald eagle)

Lch *Latimeria chalumnae* (coelacanth)

Dr *Danio rerio* (zebrafish)

Le *Leucoraja eriancea* (little skate)

Cm *Callorhinchus milii* (Australian ghostshark)

Lca *Lethenteron camtschaticum* (Arctic lamprey)

Ch *Crotalus horridus* (Timber rattlesnake)

Aa *Anguilla anguilla* (European eel)

Gm *Gadus morhua* (Atlantic cod)

Ok *Oncorhychus kisutch* (coho salmon)

**KyoT2:**

Hs *Homo sapiens* (human)

Mm *Mus musculus* (mouse)

Cl *Canis lupus* (grey wolf)

Fc *Felis catus* (domestic cat)

Lv *Lipotes vexillifer* (Chinese river dolphin)

Pc *Phascolarctos cinereus* (koala)

Md *Monodelphis domestica* (short-tailed opossum)

Hl *Haliaeetus leucocephalus* (bald eagle)

Pm *Parus major* (great tit)

Dr *Danio rerio* (zebrafish)

Ss *Salmon solar* (Atlantic salmon)

Ch *Clupea harengus* (herring)

Am *Alligator mississippiensis* (American alligator)

**Limpet:**

Dm *Drosophila melanogaster* (fruit fly)

Md *Musca domestica* (housefly)

Ag *Anopheles gamgiae* (mosquito)

Aa *Aedes aegyti* (mosquito)

Ad *Apis dorsata* (Asia honeybee)

Tc *Tribolium castaneum* (red flour beetle)

**Prickle/prickle like:**

Dm *Drosophila melanogaster* (fruit fly)

Hs *Homo sapiens* (human)

Hv *Hydra vulgaris* (fresh-water polyp)
